# Working towards safer surgery in Africa; a survey of utilization of the WHO safe surgical checklist at the main referral hospitals in East Africa

**DOI:** 10.1186/s12871-016-0228-8

**Published:** 2016-08-11

**Authors:** Isabella Epiu, Jossy Verel Bahe Tindimwebwa, Cephas Mijumbi, Francois Ndarugirire, Theogene Twagirumugabe, Edwin Rwebusiga Lugazia, Gerald Dubowitz, Thomas M. Chokwe

**Affiliations:** 1 Fogarty Global Health Fellow, University of California Global Health Institute (UCGHI), San Francisco, California USA; 2 Department of Anaesthesia, Makerere University College of Health Sciences, P.O. BOX 7072, Kampala, Uganda; 3 Mulago Hospital, Kampala, Uganda; 4 Centre Hospitalo-Universitaire de Kamenge, Bujumbura, Burundi; 5 College of Medicine and Health Sciences, University of Rwanda, Butare, Rwanda; 6 Muhimbili University of Health and Allied Sciences, Dar es Salaam, Tanzania; 7 University of California San Francisco, San Francisco, USA; 8 Department of Anaesthesia, University of Nairobi, Nairobi, Kenya

**Keywords:** WHO safe surgery checklist, Anaesthesia, Surgical safety, East Africa

## Abstract

**Background:**

Mortality from anaesthesia and surgery in many countries in Sub-Saharan Africa remain at levels last seen in high-income countries 70 years ago. With many factors contributing to these poor outcomes, the World Health Organization (WHO) launched the “Safe Surgery Saves Lives” campaign in 2007. This program included the design and implementation of the “Surgical Safety Checklist”, incorporating ten essential objectives for safe surgery. We set out to determine the knowledge of and attitudes towards the use of the WHO checklist for surgical patients in national referral hospitals in East Africa.

**Methods:**

A cross-sectional survey was conducted at the main referral hospitals in Mulago (Uganda), Kenyatta (Kenya), Muhimbili (Tanzania), Centre Hospitalier Universitaire de Kigali (Rwanda) and Centre Hospitalo-Universitaire de Kamenge (Burundi). Using a pre-set questionnaire, we interviewed anaesthetists on their knowledge and attitudes towards use of the WHO surgical checklist.

**Results:**

Of the 85 anaesthetists interviewed, only 25 % regularly used the WHO surgical checklist. None of the anaesthetists in Mulago (Uganda) or Centre Hospitalo-Universitaire de Kamenge (Burundi) used the checklist, mainly because it was not available, in contrast with Muhimbili (Tanzania), Kenyatta (Kenya), and Centre Hospitalier Universitaire de Kigali (Rwanda), where 65 %, 19 % and 36 %, respectively, used the checklist.

**Conclusion:**

Adherence to aspects of care embedded in the checklist is associated with a reduction in postoperative complications. It is therefore necessary to make the surgical checklist available, to train the surgical team on its importance and to identify local anaesthetists to champion its implementation in East Africa. The Ministries of Health in the participating countries need to issue directives for the implementation of the WHO checklist in all hospitals that conduct surgery in order to improve surgical outcomes.

## Background

Every year, tens of millions of patients worldwide suffer disabling injuries or death because of unsafe medical care [1]. Current data suggest that the death rate in high-income countries related to anaesthesia ranges from 1 in 50,000 to 1 in 200,000 [2]. Anaesthesia death rates in low and-middle-income countries (LMICs) are reportedly 100 to 1,000 times higher [3–5]. Incident reports in Thailand showed that 61 % of the incidents occurred in the operating room and were due to inexperience, lack of vigilance, inadequate pre-anaesthetic evaluation, inappropriate decisions, emergency conditions, haste, inadequate supervision, and ineffective communication [6].

A surgical checklist is a visual aid that reminds users of important issues before and after surgery. The World Health Organization’s (WHO) patient safety programme, ‘Safe Surgery Saves Lives’, developed a surgical safety checklist (SSC) as a means of improving the safety of surgical care around the world. In a multinational study involving eight hospitals from diverse economic settings, its use was shown to improve compliance with standards of care by 65 % and to reduce the death rate following surgery by nearly 50 % [7]. All sites had a reduction in the rate of major postoperative complications, with a significant reduction at three sites, one in a high-income location and two in lower-income locations [7].

Growing evidence has supported promotion of the use of checklists in several areas of healthcare and increased enthusiasm for widespread introduction of such tools in clinical practice [7–9]. The concept of using a checklist in surgical and anaesthetic practice was energized by publication of the WHO surgical safety checklist in 2008. Despite initial results demonstrating that properly-implemented surgical checklists can make a substantial difference to patient safety; however, implementation has not been straightforward. The reasons for this are varied and complex but include inconsistent leadership, lack of flexibility, and teamwork requirements, all of which may be different from current practices [10].

The East African Community is made up of low and middle-income countries [11] with limited resources for anaesthesia, including shortages of trained human resources, consumables, and equipment. It also lacks well-enforced standard operating procedures, guidelines and appropriate infrastructure for safe anaesthesia. The use of the WHO SSC, if enforced, is likely to have a greater impact on outcomes in this sub-region than in developed countries.

The aim of this study was to determine knowledge and attitudes towards the use of the surgical checklist at the national referral hospitals in East Africa. We conducted a cross-sectional survey at these hospitals to describe the challenges of anaesthesia in East Africa. As part of that survey; we collected data to document practices in using the WHO SSC, and other pre-anaesthetic surgical checklists.

## Methods

The survey was conducted from February 2013 to March 2014 in the main referral hospitals in each East Africa Community country. These were Mulago Hospital (Uganda), Kenyatta Hospital (Kenya), Muhimbili Hospital (Tanzania), Centre Hospitalier Universitaire de Kigali (Rwanda), and Centre Hospitalo-Universitaire de Kamenge (Burundi). The target population was anaesthetists working in the obstetric theatres in these national referral hospitals.

This study was part of a larger survey for the first author’s Master of Medicine in Anaesthesia dissertation, on the challenges of anaesthesia in developing countries. This looked at availability of equipment, drugs and other requirements for safe obstetric anaesthesia following the World Federation of Societies’ of Anaesthesiologists (WFSA) international guidelines for safe anaesthesia. As part of this larger survey, we collected demographic data and also asked three basic questions on the WHO surgical checklist. These were whether it was available for use, if they used it, and if not, why not. A survey tool was developed to answer these questions, and initially piloted at Mulago Hospital in Kampala.

Ethical approval was obtained from Makerere University School of Medicine Research and Ethics Committee (SOMREC), the Uganda National Council for Science & Technology Ethics Committee, and hospital ethics committees for participating hospitals including Muhimbili University of Health and Allied Services Ethics Committee, Kenyatta National Hospital/University of Nairobi Ethics and Research Committee, the University of Rwanda Faculty of Medicine Research and Ethics Committee. In Centre Hospitalo-Universitaire de Kamenge Burundi, the Dean of Medical School, Universite de Burundi considered our Ethical Approval from Makerere University and no contact with patients, and allowed us to proceed with the study. Written informed consent was obtained from all individuals participating in the study.

The sample space was calculated by proportion to size sampling. We stratified according to the number of anaesthetists available in each hospital, and the individual anaesthetists interviewed were selected by simple random sampling. We obtained a list of the physician and non-physician anaesthetists providing obstetric anaesthesia in each hospital, and contacted all of them to explain the purpose of the study and ask them to participate. We continued to make contact until we had reached the target number of participants. Once we had obtained consent from an individual, we interviewed them. An investigator administered an objective pre-set questionnaire to determine anaesthetists’ knowledge of the WHO Safe Surgical Checklist, and its availability and use at the various hospitals. Demographic data and other variables were collected from each participant including their gender, level of education, years of experience and places of work (private and public) (see Table 1). STATA 14 was used for data analysis (Statcorp, College Station, TX, USA). Results were presented in numbers/frequencies for continuous variables, and proportions for categorical variables.

**Table 1:** Distribution of baseline characteristics of study participants by country

Variable	Country					
	Mulago, Uganda	CHU Kamenge Burundi	Kenyatta, Kenya	Muhimbili Tanzania	CHU Kigali Rwanda	Overall
	*N* = 23	*N* = 5	*N* = 26	*N* = 17	*N* = 14	*N* = 85
Qualification (% respondents)						
Physician anaesthetist	1 (4)	1 (20)	14 (58)	3 (18)	2 (14)	21 (25)
Nurse anaesthetist	13 (57)	4 (80)	2 (8)	8 (47)	9 (64)	36 (43)
Clinical officer anaesthetist	8 (35)	0	5 (21)	2 (12)	0	15 (18)
Other (Anaesthesia assistant/Anaesthesia Medical Officer)	1 (4)	0	3 (13)	4 (24)	3 (21)	11 (13)
Mean age in years (SD)	43.55 (8.03)	49.00 (11.25)	41.20 (9.29)	46.40 (8.11)	35.15 (6.73)	42.40 (9.13)
Mean years of experience (SD)	11.22 (6.67)	19.40 (9.79)	11.04 (7.64)	13.29 (8.67)	11.57 (14.37)	12.16 (9.21)
Gender (%)						
Female	14 (61)	2 (40)	4 (17)	4 (24)	7 (54)	31 (38)
Male	9 (39)	3 (60)	20 (83)	13 (76)	6 (46)	51 (62)
Another place of work (%)						
Private	16 (70)	3 (100)	20 (83)	13 (76)	8 (62)	60 (75)
None	7 (30)	0	4 (17)	4 (24)	5 (38)	29 (25)

## Results

**Fig. 1:**
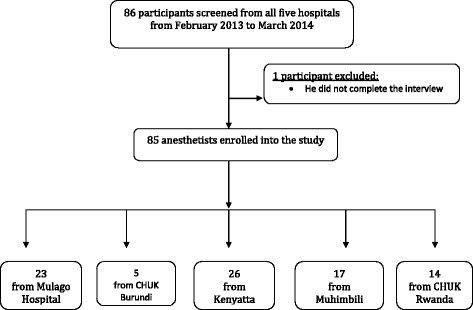
Study profile

**Fig. 2:**
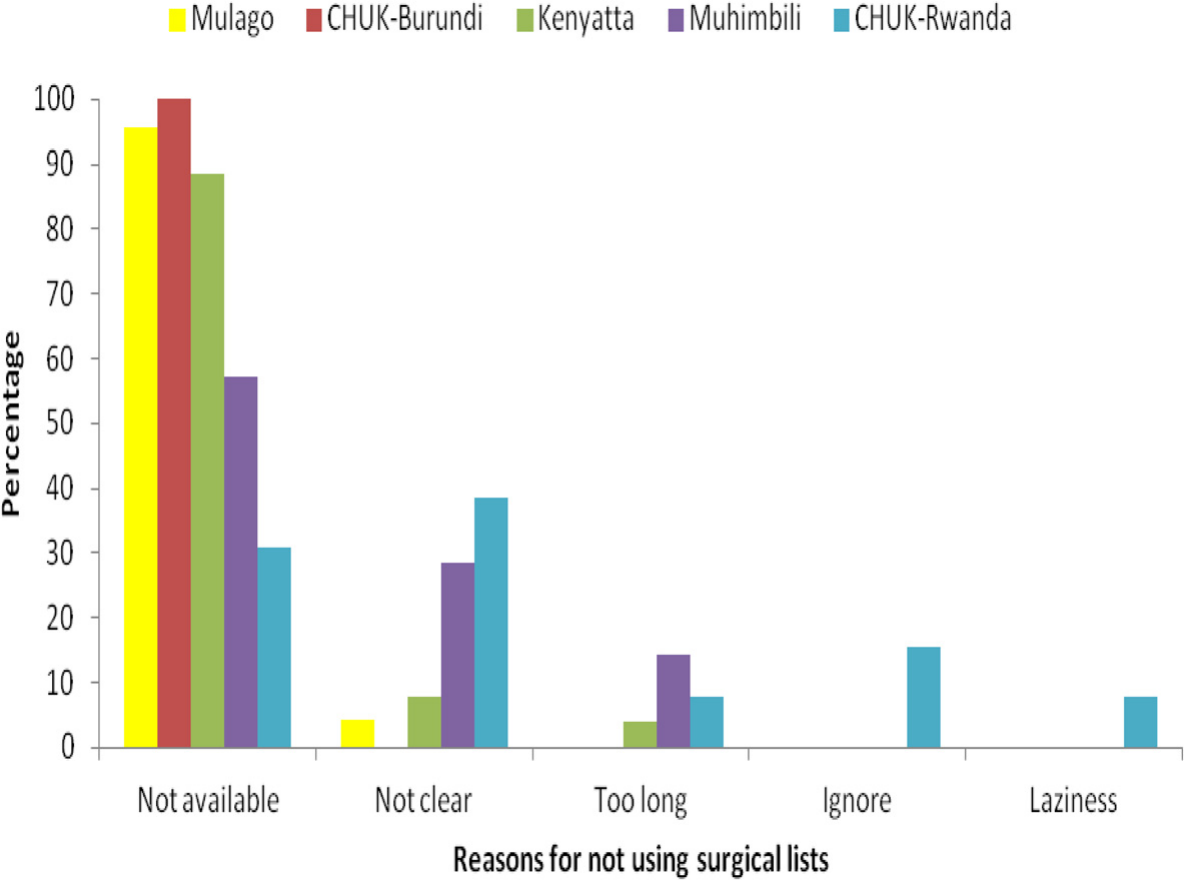
Reasons for not using the surgical checklist

Of the 86 anaesthetists contacted and interviewed, 85 responses were analysed (99 % response rate) (see Fig. 1). The participants included physician and non-physician anaesthetists working in the obstetric departments at the national referral hospitals (Table 1). One participant in Kigali consented, but was unavailable to complete the interview. A total of 58 % of participants knew about the WHO checklist, but only 25 % used it regularly (Tables 2 & 3). More than three quarters (78 %) said that the main reason the checklist was not used was because it was not available. Other reasons given were that the checklist was not clear, or was too long, or that they did not understand its purpose, were lazy or sometimes chose to ignore it (Fig. 2).

**Table 2:** Availability, knowledge and usage of the surgical checklist at 5 national referral hospitals in East Africa

Variable	Study countries					
	Mulago	CHUKBurundi	Kenyatta	Muhimbili	CHUK Rwanda	Total
Available						
Yes	1 (4)	0	3 (12)	3 (43)	9 (69)	16 (22)
No	22 (96)	5 (100)	23 (88)	4 (57)	4 (31)	58 (78)
Knowledge						
Yes	8 (35)	5 (100)	19 (73)	8 (47)	9 (64)	49 (58)
No	15 (65)	0	7 (27)	9 (53)	5 (36)	36 (42)
Usage						
Yes	0	0	5 (19)	11 (65)	5 (36)	21(25)
No	23 (100)	5 (100)	21 (81)	6 (35)	9 (64)	64 (75)

**Table 3:** Overall usage of surgical checklist at the National referral hospitals in East Africa

Usage	Frequency	Percentage (95 % CI)
Yes	21	25 (15–34)
No	64	75 (67–85)

None of the anaesthetists in Mulago (Uganda) or CHU Kamenge (Burundi) used the WHO checklist. This contrasted with Muhimbili (Tanzania), Kenyatta (Kenya), and CHU Kigali (Rwanda), where 65 %, 19 % and 36 %, respectively, used the checklist (Table 2). None of the hospitals had anyone responsible for ensuring that the surgical checklist is available in each theatre, or checking that all members of the surgical team implement it. Muhimbili had a locally designed pre-anaesthesia checklist for caesarean sections, which included a machine, drugs and airway equipment checks. However, 57 % of the anaesthetists reported that it was not generally available for use.

## Discussion

Results from 85 anaesthetists were analysed. Only 25 % regularly used the pre-anaesthetic surgical checklist, with the main reason for non-use being that it was not available. Other reasons included because the anaesthetists thought it was not clear, or too long, or they chose to ignore it.

The minimum requirements for safe anaesthesia practice include the presence of a trained provider with adequate skills, appropriate anaesthesia monitors, disposables and drugs and relevant management protocols for each level of care [12]. In 1999, the Institute of Medicine estimated that medical errors account for up to 98,000 deaths each year in the United States [13]. Failures in teamwork and communication account for 70 % of sentinel events in obstetrics [14]. Strategies to reduce errors and subsequent adverse outcomes have therefore focused on team and individual training, including simulations and drills, development of protocols, guidelines and checklists, use of information technology, and education.

Because checklists help to identify and correct preventable errors and omissions before problems arise, they are an essential step in reducing the number of adverse events by standardizing work processes. These checklists have to be tailored to the local context, and be as comprehensive as possible, but also short and clear [15]. This conclusion is supported by a 2014 systematic review of the effect of the World Health Organization surgical safety checklist on postoperative complications. Adherence to aspects of care embedded in the checklist was associated with a reduction in postoperative complications [16].

Anaesthetists are accustomed to checklists in theatre, the best known being the anaesthetic machine checklist. Safety checklists are also available for other situations, including on the ward, in the ICU (intensive care unit), and in the operating theatre. While seemingly simplistic, the evidence suggests that patients benefit from well-designed checklists used effectively. Effective implementation requires training, coaching, and a change in safety culture, with routine measurement and regular feedback of outcomes [10].

While our results may seem unusual, and specific to implementation in low and middle-income countries, a Dutch study reported that participants used the checklist only 14 out of 40 times [17]. Providers indicated that it was not used either because they did not know that there was a checklist or were already aware that the contents of the list were complete so found it unnecessary to (double) check. This study identified many of the difficulties in increasing use of checklists in the healthcare sector. A Swiss study reported a small but significant benefit when using a printed checklist as a memory tool during the sign-out process, the proportion of interventions with almost all validated items being higher than those without the memory tool (20 % vs. 0 %) [18].

Recent publications from other centres have confirmed that the sustained use of the WHO checklist improves communication and ensures the reliability of routine interventions such as antibiotic prophylaxis and thromboembolic prophylaxis [19, 20]. Although the evidence suggests that standardization of care improves patient safety, it cannot be assumed that implementation of the SSC will automatically lead to a reduction in complications. A large before–after study showed that obligatory use of the checklist was not followed by a significant effect on postoperative mortality or complication rates in Canada [21]. Studies in lower-income countries, however, have shown more marked results of using a checklist. A Tunisian study suggested that 60 % of adverse events were preventable [22]. The importance of checklist use in clinical practice is also seen in work done by a critical care specialist in Baltimore, using a checklist of steps that doctors were required to take to avoid spreading infections. It included items such as ‘wash your hands with soap’. The results of checklist use were dramatic, including a reduction in infection rate, with 43 infections avoided, eight deaths prevented and 2 million dollars saved in one hospital alone [23]. This further explains why checklists might enhance safety in hospitals.

We found that the use of the WHO SSC was very low at the study sites even though 58 % of the respondents knew about it. The response “It is not available” is perhaps a reflection of the culture and attitudes in these hospitals rather than an indication of the actual availability. It may reflect a lack of enthusiasm for checklist use because it can actually be accessed online. With the right motivation, anyone in the medical facility can print out and pin up the checklist in theatres as a reminder to the surgical team to go through each of the components for each patient. Of more concern, we noted the absence of coordinators and a perception that using the checklist may increase workload, which means that providers may be less willing to implement it, and to an extent ignore it. We believe our findings are valid, because these low-income country hospitals are faced with several other challenges, including high patient load and low resources for anaesthesia and surgery. It is, however, imperative that they use the WHO checklist, so that preventable errors are eliminated. It is essential to make sure that healthcare professionals use the checklist, and the ‘why’ and ‘how’ should therefore be communicated effectively [24].

The strengths of this study are that it was conducted at the main referral hospitals across the region, which also double as teaching hospitals for Makerere University (Uganda), Nairobi University (Kenya), Muhimbili University of Health and Allied Services (Tanzania), National University of Rwanda and University of Burundi. Anaesthetists and other theatre staff train in these hospitals, and then work across the region. Any improvements made here would therefore be spread further. Our recommendations are generalizable to all of the countries that participated in the study. We acknowledge that this study is limited by the fact that it was a cross-sectional survey of only five hospitals, which were purposively selected as representing main referral hospitals in the East African Community countries. This could have introduced a degree of selection bias, but individual anaesthetists were selected by simple random sampling.

We recommend that the health leadership in these countries is engaged and used as advocates to encourage implementation of the WHO SSC. They will need to issue directives, urging all hospitals to implement the checklist. The quality improvement systems in the hospitals need to sensitize all members of the surgical team to the evidence on improved surgical outcomes with use of the WHO SSC, and draw up a strategic plan to operationalise its implementation. This could include training anaesthetists and other members of the surgical team on why and how, and building a team of local champions to coordinate and implement its use. To address the perception of increased work, the WHO SSC could be pinned up in all theatres as a memory tool for the surgical team to use, without having to write anything down.

Our study only focused on the knowledge and attitudes towards the use of the WHO SCC among anaesthetists. Further studies are needed to ensure the checklist is available to other members of the surgical team, and to compare attitudes to and knowledge about its use across the team.

## Conclusions

Implementation of the WHO surgical checklist has the potential to reduce surgical complications in both developed and developing countries. Current data suggest that the effect is likely to be greater in lower-income countries, where resources are more limited and gaps more likely to occur. Our study shows that the checklist is inconsistently used across the East African Community even where local modifications have been developed. We conclude that there is an urgent need to make it available and to train the whole perioperative team on its importance and implementation. The Ministries of Health in developing countries need to engage quality improvement teams and issue directives for the implementation of the WHO SSC in all hospitals that conduct surgery.

## Abbreviations

CHU, centre hospitalier universite; ECG, electrocardiogram; ICU, intensive care unit; SSC, safe surgical checklist; WFSA, World Federation of Societies of Anaesthesiologists; WHO, World Health Organization

## References

[1] The Research Priority Setting Working Group of the World Alliance for Patient Safety. Summary of the evidence on patient safety: implications for research. World Health Organization. 2008. http://www.who.int/iris/handle/10665/43874. Accessed Aug 2016.

[2] Gibbs N, Rodoreda P (2005). Anaesthetic mortality rates in Western Australia 1980–2002. Anaesth Intensive Care.

[3] Hansen D, Gausi S, Merikebu M (2000). Anaesthesia in Malawi: complications and deaths. Trop Dr.

[4] Hornbein TF (1986). The setting of standards of care. JAMA.

[5] Maman A, Tomta K, Ahouangbevi S, Chobli M (2005). Deaths associated with anaesthesia in Togo, West Africa. Trop Dr.

[6] Charuluxananan S, Suraseranivongse S, Jantorn P, Sriraj W, Chanchayanon T, Tanudsintum S (2011). Multicentered study of model of anesthesia related adverse events in Thailand by incident report (The Thai Anesthesia Incidents Monitoring Study): results. J Med Assoc Thail.

[7] Haynes AB, Weiser TG, Berry WR, Lipsitz SR, Breizat A-HS, Dellinger EP (2009). A surgical safety checklist to reduce morbidity and mortality in a global population. N Engl J Med.

[8] Blot K, Bergs J, Vogelaers D, Blot S, Vandijck D (2014). Prevention of central line-associated bloodstream infections through quality improvement interventions: a systematic review and meta-analysis. Clin Infect Dis.

[9] de Vries EN, Prins HA, Crolla RM, den Outer AJ, van Andel G, van Helden SH (2010). Effect of a comprehensive surgical safety system on patient outcomes. N Engl J Med.

[10] Walker IA, Reshamwalla S, Wilson IH (2012). Surgical safety checklists: do they improve outcomes?. Br J Anaesth.

[11] World Bank. Low–Middle Income countries [http://data.worldbank.org/country] Cited Aug 2016, Visited Aug 2016 (Kenya, Uganda, Tanzania, Rwanda, & Burundi) LMC.

[12] Dyer RA, Reed AR, James MF (2010). Obstetric anaesthesia in low-resource settings. Best Pract Res Clin Obstetrics Gynaecol.

[13] Kohn L, Corrigan, JM, Donaldson, MS (Eds). Committee on Quality of Health Care in America, Institute of Medicine. To err is human: building a safer health system. Washington, DC, National Academy Press; 1999.

[14] JCAHO: Joint Commission on Accreditation of Healthcare Organizations JCAHO sentinel event alert #30. 2004.

[15] Vandijck D, Bergs J (2014). The WHO surgical safety checklist : an innovative or an irrelevant tool?. Acta Anaesth Belg.

[16] Bergs J, Hellings J, Cleemput I, Zurel Ö, De Troyer V, Van Hiel M (2014). Systematic review and metaanalysis of the effect of the World Health Organization surgical safety checklist on postoperative complications. Br J Surg.

[17] Hartskeerl M. Patient safety check: can a checklist enhance patient safety via the collaboration between doctors and nurses? Erasmus Universiteit. 2013.

[18] Cullati S, Le Du S, Rae A-C, Micallef M, Khabiri E, Ourahmoune A (2013). Is the Surgical Safety Checklist successfully conducted? An observational study of social interactions in the operating rooms of a tertiary hospital. BMJ Qual Safety.

[19] Dahl AW, Robertsson O, Stefánsdóttir A, Gustafson P, Lidgren L (2011). Timing of preoperative antibiotics for knee arthroplasties: Improving the routines in Sweden. Patient safety in surgery.

[20] Berrisford RG, Wilson IH, Davidge M, Sanders D (2012). Surgical time out checklist with debriefing and multidisciplinary feedback improves venous thromboembolism prophylaxis in thoracic surgery: a prospective audit. Eur J Cardiothorac Surg.

[21] Urbach DR, Govindarajan A, Saskin R, Wilton AS, Baxter NN (2014). Introduction of surgical safety checklists in Ontario, Canada. N Engl J Med.

[22] Letaief M, El Mhamdi S, El-Asady R, Siddiqi S, Abdullatif A (2010). Adverse events in a Tunisian hospital: results of a retrospective cohort study. Int J Qual Health Care.

[23] Atul G (2011). The checklist manifesto: how to get things right.

[24] Conley DM, Singer SJ, Edmondson L, Berry WR, Gawande AA (2011). Effective surgical safety checklist implementation. J Am Coll Surg.

